# Prevalence and Identification of *Burkholderia pseudomallei* and Near-Neighbor Species in the Malabar Coastal Region of India

**DOI:** 10.1371/journal.pntd.0004956

**Published:** 2016-09-15

**Authors:** Bhavani V. Peddayelachagiri, Soumya Paul, Sowmya Nagaraj, Madhurjya Gogoi, Murali H. Sripathy, Harsh V. Batra

**Affiliations:** 1 Microbiology Division, Defence Food Research Laboratory, Karnataka, India; 2 Centre for Biotechnology and Bioinformatics, Dibrugarh University, Dibrugarh, Assam, India; University of Tennessee, UNITED STATES

## Abstract

Accurate identification of pathogens with biowarfare importance requires detection tools that specifically differentiate them from near-neighbor species. *Burkholderia pseudomallei*, the causative agent of a fatal disease melioidosis, is one such biothreat agent whose differentiation from its near-neighbor species is always a challenge. This is because of its phenotypic similarity with other *Burkholderia* species which have a wide spread geographical distribution with shared environmental niches. Melioidosis is a major public health concern in endemic regions including Southeast Asia and northern Australia. In India, the disease is still considered to be emerging. Prevalence surveys of this saprophytic bacterium in environment are under-reported in the country. A major challenge in this case is the specific identification and differentiation of *B*. *pseudomallei* from the growing list of species of *Burkholderia* genus. The objectives of this study included examining the prevalence of *B*. *pseudomallei* and near-neighbor species in coastal region of South India and development of a novel detection tool for specific identification and differentiation of *Burkholderia* species. Briefly, we analyzed soil and water samples collected from Malabar coastal region of Kerala, South India for prevalence of *B*. *pseudomallei*. The presumptive *Burkholderia* isolates were identified using *recA* PCR assay. The *recA* PCR assay identified 22 of the total 40 presumptive isolates as *Burkholderia* strains (22.72% and 77.27% *B*. *pseudomallei* and non-*pseudomallei Burkholderia* respectively). In order to identify each isolate screened, we performed *recA* and 16S rDNA sequencing. This two genes sequencing revealed that the presumptive isolates included *B*. *pseudomallei*, non-*pseudomallei Burkholderia* as well as non-*Burkholderia* strains. Furthermore, a gene termed D-beta hydroxybutyrate dehydrogenase (*bdha*) was studied both *in silico* and *in vitro* for accurate detection of *Burkholderia* genus. The optimized *bdha* based PCR assay when evaluated on the *Burkholderia* isolates of this study, it was found to be highly specific (100%) in its detection feature and a clear detection sensitivity of 10 pg/μl of purified gDNA was recorded. Nucleotide sequence variations of *bdha* among interspecies, as per *in silico* analysis, ranged from 8 to 29% within the target stretch of 730 bp highlighting the potential utility of *bdha* sequencing method in specific detection of *Burkholderia* species. Further, sequencing of the 730 bp *bdha* PCR amplicon of each *Burkholderia* strain isolated could differentiate the species and the data was comparable with *recA* sequence data of the strains. All sequencing results obtained were submitted to NCBI database. Bayesian phylogenetic analysis of *bdha* in comparison with *recA* and 16S rDNA showed that the *bdha* gene provided comparable identification of *Burkholderia* species.

## Author Summary

With the growing list of *Burkholderia* species, which represents a genus of bacteria with economical, health and biowarfare importance, the need for specific and efficient detection systems is also growing. *Burkholderia pseudomallei* is reported to be endemic in India. However, limited prevalence studies have been conducted in the country to determine the presence of this pathogen in environment. The high level of phenotypic and genotypic similarities of this bacterium with other species of the genus stands as a difficult challenge to differentiate them. Several molecular detection systems have been reported in this field, with a range of identification potential restricted to *Burkholderia cepacia* complex. The present study addresses the former mentioned challenges by performing a surveillance study in Malabar coastal region of South India wherein we encountered 22 strains of *Burkholderia*. Furthermore, we determined the utility of a novel gene for specific detection of the whole *Burkholderia* genus by developing a novel *bdha* based PCR assay. The study design includes extensive bioinformatic analyses and comparative genomics for the development of the described compound detection tool. The study also includes the evaluation of the genus-specific gene of its efficiency in specifically identifying the *Burkholderia* species by nucleotide sequencing based method. The novel genus-specific gene is also evaluated for its role in phylogeny of *Burkholderia* species in comparison with that of earlier reported *recA* and 16S rDNA genes.

## Introduction

In India, melioidosis is considered as an emerging disease [1]. Though melioidosis cases are considered to be significantly under-reported in India, few studies suggest that the disease is completely prevalent in India [2, 3]. Some clinical cases of melioidosis incidences are being documented from states of Karnataka, Tamilnadu, Pondicherry, Kerala, Maharashtra, Orissa, Assam, West Bengal and Tripura [4]. Nevertheless, very fewer surveys, in the country, have been undertaken to study the environmental distribution of *B*. *pseudomallei*, the causative agent of melioidosis. This could be due to factors such as limited laboratory support and/or low clinical suspicion in some areas. Howbeit, this information is highly useful in determination of the disease transmission pathway and epidemiology [5].

Isolation and culturing is recommended as the “gold standard” method of *B*. *pseudomallei* identification in all reference laboratories till date [6]. Gram staining, antibiotic susceptibility testing, specific biochemical tests such as maltose acidification, esculin hydrolysis, arginine dihydrolase test and colonial characteristics on differential Ashdown agar are also used for confirmation of this bacterium [7, 8]. In this scenario, the availability of a pure culture is critical. The high degree of phenotypic and genotypic similarity presented by the members of *Burkholderia* genus stands as a major challenge in differentiating this pathogen from near-neighbor *Burkholderia* species [9]. In such cases, direct identification methods are most beneficial.

Of the 80 and above *Burkholderia* species [10], whole genome sequence based information for more than 35 species has been made available to the scientific community [11]. This genetic information is being used to understand the taxonomic position of these bacteria and also to differentiate the species. Comprehensive phylogenetic analysis of *Burkholderia* species based on 16S rDNA [10, 12], *recA* [13–16] and *acdS* [17] genes has coherently compartmentalized them into two main lineages–(1) *Burkholderia* species with pathogenic properties and (2) Non-pathogenic species with positive association with plants. Several DNA based methods for detection of *Burkholderia* species of biowarfare agents namely *B*. *pseudomallei* and *B*. *mallei* have also been reported. The methods include PCR-RFLP [18, 19], PCR followed by sequencing [15], multiplex PCRs [20], RT-PCRs [21–23] and so on. In case of *recA*, sequence polymorphism provides clear distinction between genomovars of BCC [13–15]. However, as a limitation, *recA* based pyrosequencing assay was not able to differentiate *B*. *multivorans* strains of *B*. *cepacia* complex (BCC) from *B*. *ambifaria* [24]. The former methods, though sensitive, are time consuming (eg. PCR-RFLP) and are not cost effective (eg. RT-PCR). Additionally, they require skilled personnel for result interpretation. In case of conventional PCRs, the differential identification is difficult in interpretation, as strains of a single species give different amplicon sizes [20]. Since *Burkholderia* species include bacteria of multi-host pathogens related to emerging infectious diseases as well as biotechnological importance, need for a promising, rapid, robust, easily interpretable and cost effective detection tool for specific identification of individual species is ascending.

The western coastal region of India, with an annual rainfall of about 300 cm, agriculture as the major occupation and a high population of diabetics, is predicted for endemicity of melioidosis [25]. Clinico-epidemiological studies reported from this region reveal the prevalence of melioidosis among patients [25, 26, 27]. This disease occurs among susceptible hosts predominantly during wet season and *B*. *pseudomallei* increases in number and survival in environment during rainfall and with the rise in water table level [15, 28]. Canvassing soil for occurrence of *Burkholderia* is routinely followed in endemic regions since it is the common dwelling place for *Burkholderia* and novel *Burkholderia* species are often encountered [9, 29]. However, no environmental survey has been undertaken, to the best of our knowledge. With this background, this work aimed to study the prevalence of *B*. *pseudomallei* in soil and water samples collected from Malabar coastal region of Kerala, South India during rainy and winter season. To achieve this objective, we employed conventional isolation method followed by *recA* and 16S rDNA sequencing method to identify the *Burkholderia pseudomallei* strains isolated from environmental samples. During this study, we encountered *Burkholderia pseudomallei*, non-*pseudomallei Burkholderia* and non-*Burkholderia* strains and were identified by *recA* and 16S rDNA sequencing scheme. Furthermore, we developed a novel *bdha* PCR assay to include in the routine *recA* and 16S rDNA sequencing scheme for better and accurate identification of *Burkholderia* species. Further, the evolutionary role of *bdha* among the *Burkholderia* species was studied by performing Bayesian phylogenetic analysis in comparison with that of *recA* and 16S rDNA genes.

## Methods

### Bacterial strains and media

*Burkholderia pseudomallei* NCTC 10274 was received from Defence Research Development and Organization and other reference strains listed in Table 1 were procured from ATCC (USA), MTCC (Chandigarh, India) and NCIM (Imtech, Pune, India). All bacterial strains and isolates used in this study were maintained at -80°C in 15% glycerol stocks for future use. Ashdown’s selective medium was used for isolation of *Burkholderia*. Unless mentioned, all bacterial cultures were cultivated using trypticase soy broth (TSB) and trypticase soy agar (TSA). ASH was prepared as described previously (Ashdown 1979) using individual dehydrated media components and salts (Himedia, India). Antibiotics were used at a final concentration of 4 mg/ml for gentamicin and 100 mg/ml for ampicillin.

**Table 1:** Bacterial strains used in this study and their respective identification profile.

Serial number	Bacteria	Source	*recA* PCR	*bdha* PCR
1	*Burkholderia pseudomallei* NCTC 10274	NCTC	+	+
2	*Burkholderia pseudomallei* DFRL001	Soil	+	+
3	*Burkholderia pseudomallei* DFRL002	Soil	+	+
4	*Burkholderia pseudomallei* DFRL003	Soil	+	+
5	*Burkholderia pseudomallei* DFRL004	Soil	+	+
6	*Burkholderia pseudomallei* DFRL005	Soil	+	+
7	*Burkholderia cepacia* MTCC 1617	MTCC	+	+
8	*Burkholderia cepacia* DFRL011	Soil	+	+
9	*Burkholderia cepacia* DFRL012	Soil	+	+
10	*Burkholderia cepacia* DFRL013	Soil	+	+
11	*Burkholderia cepacia* DFRL014	Soil	+	+
12	*Burkholderia cenocepacia* DFRL-Bcc01	Soil	+	+
13	*Burkholderia cenocepacia* DFRL015	Soil	+	+
14	*Burkholderia cenocepacia* DFRL016	Soil	+	+
15	*Burkholderia cenocepacia* DFRL017	Soil	+	+
16	*Burkholderia cenocepacia* DFRL018	Soil	+	+
17	*Burkholderia cenocepacia* DFRL019	Soil	+	+
18	*Burkholderia cenocepacia* DFRL020	Soil	+	+
19	*Burkholderia cenocepacia* DFRL021	Water	+	+
20	*Burkholderia vietnamiensis* ATCC 55792	ATCC	+	+
21	*Burkholderia vietnamiensis* DFRL022	Soil	+	+
22	*Burkholderia vietnamiensis* DFRL023	Soil	+	+
23	*Burkholderia vietnamiensis* DFRL024	Water	+	+
24	*Burkholderia diffusa* DFRL-Bd01	Soil	+	+
25	*Burkholderia diffusa* DFRL025	Soil	+	+
26	*Burkholderia diffusa* DFRL026	Water	+	+
27	*Burkholderia anthina* DFRL027	Water	+	+
28	*Burkholderia gladioli* MTCC 1888	MTCC	+	+
29	*Comamonas testosteroni* DFRL1001	Water	-	-
30	*Chromobacterium violaceum* DFRL1002	Water	-	-
31	*Stenotrophomonas maltophilia* DFRL1017	Soil	-	-
32	*Achromobacter xylosoxidans* DFRL1023	Soil	-	-
33	*Achromobacter ruhlandii* DFRL1024	Soil	-	-
34	*Pseudomonas stutzeri* DFRL567	Soil	-	-
35	*Pseudomonas aeruginosa* ATCC 27853	ATCC	-	-
36	*Proteus mirabilis* MTCC 3310	MTCC	-	-
37	*Citrobacter freundii* ATCC 8090	ATCC	-	-

* ATCC—American Type Culture Collection, Manassas, USA

** NCTC—National Collection of Type Cultures, Salisbury, UK

*** MTCC—Microbial Type Culture Collection, Chandigarh, India

**** NCIM—National Collection of Industrial Microorganisms, Pune, India

### *Burkholderia* isolation

Malabar coastal region of South India was selected for isolation of *Burkholderia* from soil and water samples. Thirty soil samples from rubber plantations in Malabar region and 12 water samples from river banks of Kerala Backwaters were collected during June (rainy season), October and November (winter season). Briefly, 20–30 g of each soil sample from selected sites was collected from depths of 10 cm and 30 cm using sterile spatula in sterile pouch having zip locks. Approximately 1 l of each water sample was collected from 50 cm depth of river banks in sterile glass containers. Precautions were taken to prevent cross-contamination during sampling. The collected samples were further transported to Defence Microbiology laboratory and stored at 4°C until examination for prevalence of *Burkholderia*. For sample preparation, 5 g of soil or 5 ml of water was aliquoted into a sterile container and 30 ml of sterile distilled water was added. The suspension was shaken vigorously for 1 min and allowed to settle for 5–10 min. From the supernatant fluid thus obtained, 2 ml was transferred into 18 ml of Ashdown's selective enrichment broth (ASB) [30] and incubated for enrichment at 37°C for 48 h. After incubation, 100 μl of the broth was spread onto Ashdown’s selective enrichment agar (ASA) [7] and incubated at 37°C for 48 h. Presumptive isolates obtained were streaked onto trypticase soy agar (TSA), incubated at 37°C for 24 h and purified. All purified isolates were stored in trypticase soy broth (TSB) containing 15% glycerol at -80°C for future use.

### DNA preparation

Total genomic DNA (gDNA) from bacterial strains and isolates used in this study was isolated according to a protocol described elsewhere [31]. Each DNA sample thus prepared was spectrophotometrically quantified using Nanodrop 2000 spectrophotometer (Thermo Scientific Nanodrop, USA) and stored frozen at −20°C until use.

### PCR confirmation

The presumptive *Burkholderia* isolates recovered from the soil and water samples were confirmed using *recA* PCR assay, wherein gDNA from reference strains of *Burkholderia* including *B*. *pseudomallei*, *B*. *mallei*, *B*. *cepacia* and *B*. *gladioli* were used as positive control. The PCR assay was performed using BUR3 and BUR4 primers (Table 2) as described elsewhere [15].

**Table 2:** Primers used in this study.

Target gene		Sequence (5’– 3’)	Amplicon size (~ bp)	References
*recA*	BUR3	GA(AG)AAGCAGTTCGGCAA	385	[15]
	BUR4	GAGTCGATGACGATCAT		
*recA*	BUR1	GATCGA(AG)AAGCAGTTCGGCAA	869	[15]
	BUR2	TTGTCCTTGCCCTG(AG)CCGAT		
16S rDNA	27f	AGAGTTTGATCATGGCTCAG	1491	[35, 36]
	1492r	TACCTTGTTACGACTT		
*bdha* (BPSS0017)	*bdha*-f	ACCAGCCGTGGCTGACGAC	730	This study
	*bdha*-r	GCGGCATCGGCAAGGAAATC		

### *bdha in silico* analysis

An initial *in silico* nucleotide search for highly conserved *Burkholderia* genus specific genes with marked level of interspecies variation was conducted using whole genome sequence of *B*. *pseudomallei* strain K96243 (accession no. BX571966) available on National Center for Biotechnology Information (NCBI) database (http://www.ncbi.nlm.nih.gov) as reference. Care was taken to screen evolutionary conserved, fundamentally/metabolically important and versatility genes for this purpose. Accordingly, nucleotide sequences of oxidoreductases family, chaperones and structural protein genes were used as queries to determine specific sequences within the genus of *Burkholderia*. For each matching nucleotide sequence, *in silico* coverage analysis was done at two taxonomic levels of *Burkholderia*—genus and family Burkholderiaceae; using online BLASTN analysis (http://blast.ncbi.nlm.nih.gov/Blast.cgi). In this step, sequences showing near 100% coverage of query sequence and significant expect (e) value of < 10^-100^ were selected. Promising nucleotide sequences were further edited according to the BioEdit v7.2.5 (http://www.mbio.ncsu.edu/bioedit/bioedit.html) software default parameters and analyzed for the distribution of conserved regions and variation among the interspecies of the genus using the Entropy (H_x_) plot option [32]. Of the sequences encountered, 3-hydroxybutyrate dehydrogenase (*bdha*) (BPSS0017) was selected as genus-specific gene target by analyzing for the unique detection specificity within the genus required for the present study. Pairwise progressive sequence alignment of *bdha* nucleotide sequence of *Burkholderia* and non-*Burkholderia* species, which were retrieved from NCBI, was performed using Clustal X2. Entropy plot for *bdha* was generated using BioEdit v7.2.5. The identity scores for the *bdha* sequence of *Burkholderia* species were obtained by the use of GAP program of GCG Wisconsin package (http://www.biology.wustl.edu/gcg/before_you_begin.html).

### *bdha* PCR assay development

After *in silico* nucleotide analysis, whole-genome sequence of *B*. *pseudomallei* strain K96243 was referred for designing primers for specific amplification of the *bdha* gene. Gene Runner 3.0 software (http://www.generunner.net/) was used for designing and evaluating the primer pair. Care was taken so that the primer is situated in the blocks of highly conserved regions showing nearly 100% conservation in *bdha* and so as to establish PCR-sequencing based differentiation among the interspecies of *Burkholderia*. Table 2 lists the primers designed with respective oligonucleotide sequence and amplicon size. Each 20 μl PCR reaction contained 1.25 U *Taq* polymerase (Sigma, India), 50 μM of each deoxynucleotide triphosphate (Fermentas, India), 1x PCR buffer, 2.0 mM MgCl_2_, 0.6 pmol of each oligonucleotide primer, and 50 ng of template DNA. Thermal cycling was carried out in Master cycler-Pro thermal cycler (Eppendorf, Germany) using the designed primers with an initial denaturation for 4 min at 94°C followed by 30 cycles of denaturation for 30 s at 94°C, annealing for 30 s at 58°C, and extension at 72°C for 45 s, with a final 8 min extension at 72°C. Approximately 5 μl of each PCR product was visualized by agarose gel (2% w/v) electrophoresis.

### Specificity and sensitivity determination

To determine the authenticity of the *bdha* PCR assay in terms of detection specificity, PCR was performed as above using genomic DNA of both *Burkholderia* and non-*Burkholderia* bacteria listed in Table 1 as template. Each strain was evaluated for reactivity at least twice. The sensitivity of the optimized end-point PCR format was assayed using *B*. *pseudomallei* ATCC 23343. To achieve this, gDNA was serially diluted tenfold from 100 ng/μl to 1 pg/μl using double distilled water and PCR was performed as mentioned above with 1 μl of DNA dilutions as template. Approximately 5 μl of each PCR product was visualized by agarose gel (2% w/v) electrophoresis.

### Sequencing analysis

For both *Burkholderia* and non-*Burkholderia* isolates that were isolated in this study, *recA*, *bdha* and 16S rDNA gene sequencing was performed to confirm the organism identity. Respective primers used for PCR amplifying the three genes from each isolate are listed in Table 2. Sequencing reactions were prepared using Applied Biosystems Big Dye Terminator ready reaction mix version 3.1 as per manufacturer’s instructions and capillary electrophoresis was run in Applied Biosystems ABI-Prism 3100 genetic analyzer system using Applied Biosystems Performance Optimized Polymer 6 (POP-6). Resultant raw sequence data obtained from both template and anti-sense strands of the PCR products were aligned to derive consensus sequence using the CAP contig assembly program of the BioEdit software v7.2.5. Consensus sequences were analyzed using Basic Local Alignment Search Tool (BLASTN) (www.ncbi.nlm.nih.gov) to establish the correct organism identity. The sequences were analyzed for chimera as described elsewhere [33].

### Nucleotide sequence accession numbers

*bdha*, *recA* and 16S rDNA gene sequence alignments of all *Burkholderia* isolates and reference strains were submitted to GenBank under the accession numbers KP190932 to KP190939, KP638773 to KP638776, KU84505 to KU84507, KU749955 to KU749969 and KU749970 to KU749990.

### Phylogenetic analysis

Bayesian analysis was conducted using the *bdha*, *recA* and 16S rDNA gene sequences to determine the rate of diversity within *Burkholderia* genus, as depicted by each gene. The nucleotide sequences of *Burkholderia* species of this study were also involved in this exercise (S1 Supporting Information). MrBayes version v3.2 (MCMC package) was used for this purpose [34]. The diagnostic frequency was set for 5,000 generation. The default run length was 50,000 or until the standard deviation of split frequencies was below 0.05. The resultant phylogram of each gene examined was drawn using FigTree v.1.4.2 software [http://tree.bio.ed.ac.uk/software/figtree/].

## Results

### Isolates recovered

Of the total 40 samples screened, 19 (63.33%) and 5 (41.66%) soil and water samples respectively resulted in bacterial growth on ASH agar (Table 1). All 24 samples exhibited bacteria with multiple colony morphologies. Forty colonies (approx. 1–3 colonies from each sample based on the colony morphology) were non-randomly chosen as representatives of colony morphologies observed and purified from ASH agar. The presumptive isolates thus obtained were consistent in their respective growth feature, upon purification and repeated sub-culturing on ASH medium. Colony morphology of the isolates range from typical wrinkled *B*. *pseudomallei* colonies, to variable colonies in terms of size, texture, appearance, color and growth rate. The isolates recovered included *Burkholderia* species namely, *B*. *pseudomallei*, *B*. *thailandensis*, *B*. *cepacia*, *B*. *cenocepacia*, *B*. *diffusa*, *B*. *gladioli*, *B*. *anthina* and *B*. *vietnamiensis* and bacteria of other genera including *Comamonas testosteroni*, *Chromobacterium violaceum*, *Stenotrophomonas maltophilia*, *Achromobacter xylosoxidans*, *Achromobacter ruhlandii*, *Pseudomonas stutzeri*, *Klebsiella pneumoniae*, *Enterobacter cloacae*, *Pandoraea* sp. and *Chryseobacterium* sp.

### PCR identification

The recovered presumptive isolates were screened for *Burkholderia* genus by employing *recA* based specific PCR targeting 385 bp of *recA* gene. This resulted in identifying 22 of the 40 presumptive isolates as *Burkholderia* species (Table 1). The PCR analysis was repeated thrice and the results were found to be unambiguously reproducible. As a test of sensitivity, gDNA from 18 PCR-negative isolates was conventionally isolated and evaluated by PCR. In this repetitive PCR screening, all 18 isolates were subsequently negative. On the other hand, all 22 *recA* positive isolates were concurrently found to be *Burkholderia* when re-tested for PCR assay using conventionally isolated gDNA.

### Conservation and variation analysis of *bdha*

**Fig 1:**
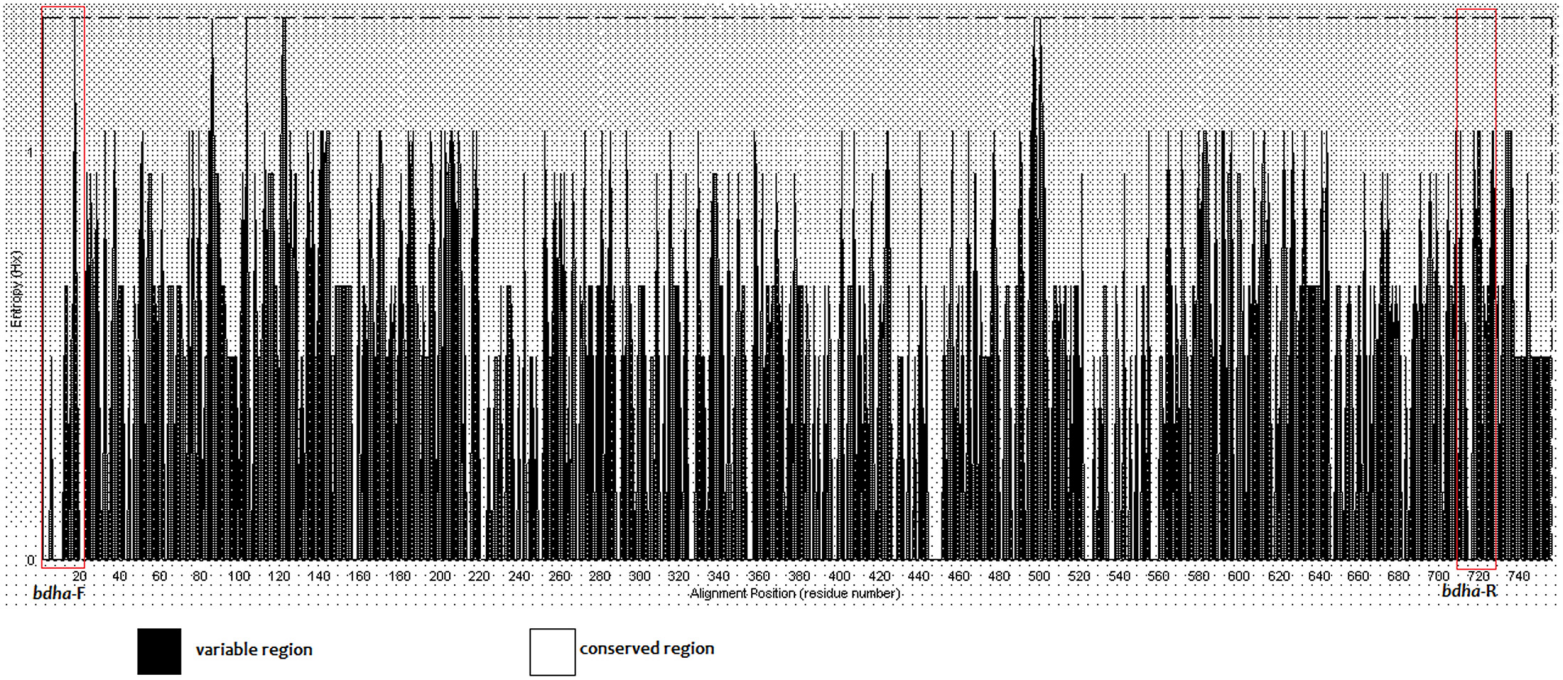
Analysis of *bdha* divergence among *Burkholderia* species. The 786 bp length *bdha* of *Burkholderia* species available in NCBI database were analyzed in Bioedit software for conservation and variation. “A” region is more variable portion and “B” is more conserved portion with the gene.

**Fig 2:**
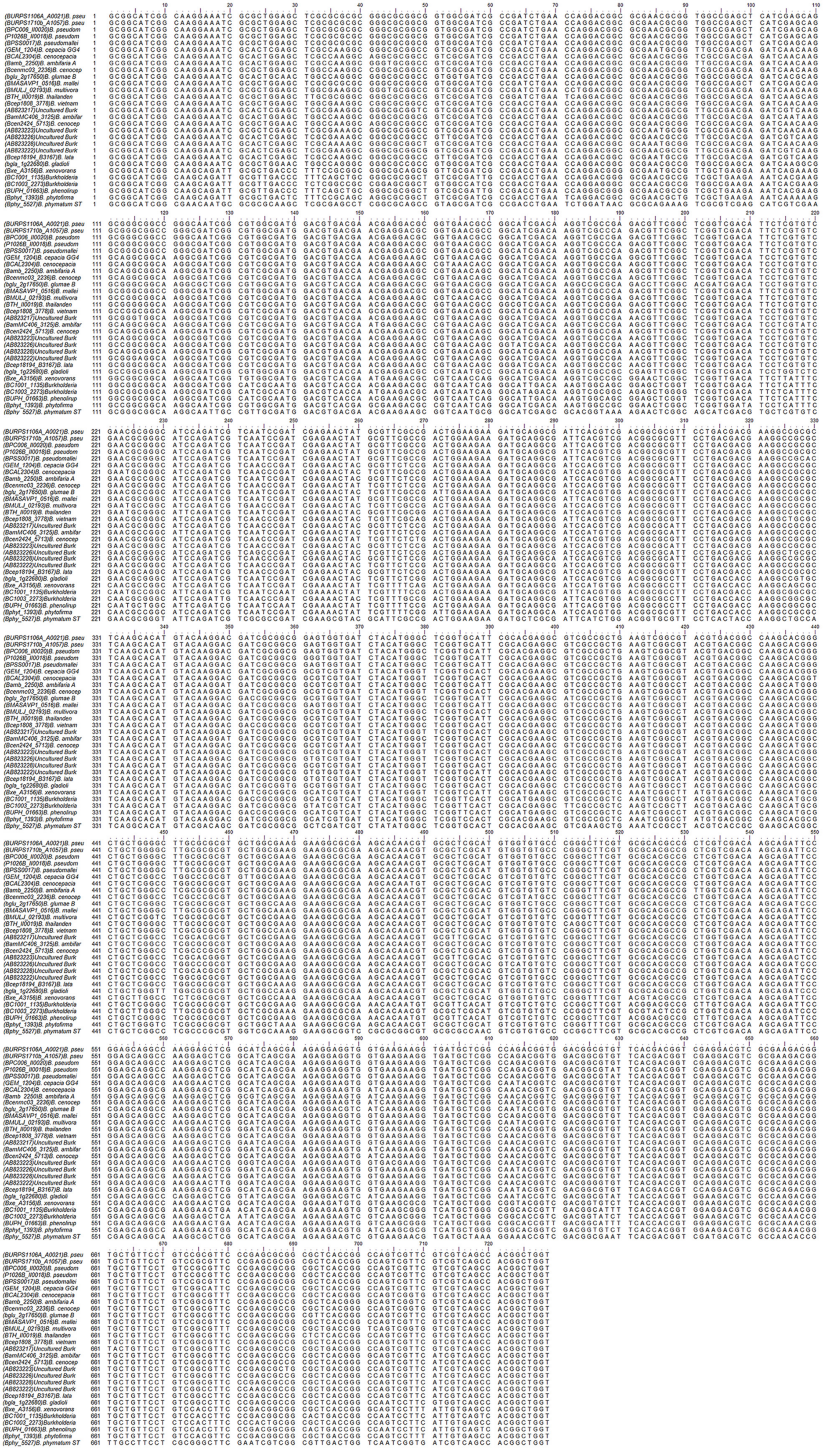
Multiple sequence alignment of 730 bp stretch of *bdha* nucleotide sequences of *Burkholderia* species. The nucleotide stretch of *bdha* targeted in PCR amplification for specific detection of *Burkholderia* species were aligned using Clustal Omega. The alignment reveals the conservedness of the gene stretch at the extremes along with the variations throughout the stretch.

A BLASTN analysis of *bdha* sequence of *B*. *pseudomallei* K96243 strain resulted in alignment of the gene against almost all the sequenced *Burkholderia* species and strains as well as *Achromobacter xylosoxidans* with significant expect (e) value of < 10^-100^. Insignificant e-value match (approx. 9 x 10^-117^) was recorded against newly added species of *Burkholderia—B*. *phymatum*, along with *Ralstonia solanacearum*, *Chromobacterium violaceum*, *Pseudomonas dinitrificans*, *Thiomonas intermedia*, *Hyphomicrobium* species, *Janthinobacterium agaricidamnosum*, *Actinoplanes missouriensis* and *Kitasatospora setae*. Further *in silico* analysis of *bdha* nucleotide sequence for conservation and homology among *Burkholderia* species revealed a characteristic pattern of the arrangement of conservative regions. These regions were found to be distributed in a mosaic pattern within the variable regions (Fig 1). These variation regions were found to be corresponding single nucleotide mutation points throughout the gene with the *Burkholderia* genus as noticed in ClustalX2 alignment (Fig 2). This characteristic genetic distinction of *bdha* within the *Burkholderia* genus encouraged us for development of *Burkholderia* genus-specific PCR assay.

From the *in silico* nucleotide sequence analysis using GAP program, it was evident that the level of sequence variations among *Burkholderia* inter-species was rather high due to the sequence polymorphisms; ranging from 8% to 29% (Table 3). To explain, the variations were—for *B*. *pseudomallei* (i) 8–9% with *B*. *cepacia*, *B*. *cenocepacia*, *B*. *ambifaria*, *B*. *multivorans*, *B*. *vietnamiensis* and uncultured *Burkholderia* species HBadh-2, HBadh-7, HBadh-8, HBadh-13, and HBadh-15, (ii) 10% with *B*. *gladioli*, (iii) 15–17% with *B*. *phytofirmans*, *B*. *xenovorans* and *Burkholderia* sp. CCGE1001 and CCGE1003, (iv) 24% with *B*. *phymatum*, (v) 7% between *B*. *ambifaria* and *B*. *multivorans*, (vi) 21–25% for *B*. *phymatum* with all *Burkholderia* species. However, highest rate of similarity ranging from 96 to 99% was noticed among *B*. *pseudomallei*, *B*. *mallei* and *B*. *thailandensis*. Additionally intra-species variations ranged from 0 to 0.1%. The higher level of sequence variations in *bdha* sequence among *Burkholderia* species confers more possibilities for confirmative distinction on nucleotide sequencing platform.

**Table 3:** *bdha* nucleotide sequences identity between the *Burkholderia* species.

Species no.	Species name	% Identity with species no.																												
		1	2	3	4	5	6	7	8	9	10	11	12	13	14	15	16	17	18	19	20	21	22	23	24	25	26	27	28	29
**1**	***B*. *pseudomallei* K96243**	ID	0.997	1	0.994	0.994	0.914	0.908	0.909	0.914	0.912	0.995	0.91	0.961	0.925	0.906	0.906	0.906	0.906	0.905	0.909	0.909	0.909	0.895	0.834	0.821	0.828	0.82	0.842	0.755
**2**	***B*. *pseudomallei* 1106a**	0.997	ID	0.997	0.997	0.997	0.914	0.908	0.909	0.914	0.914	0.998	0.91	0.961	0.925	0.906	0.906	0.906	0.906	0.905	0.909	0.909	0.909	0.895	0.831	0.821	0.828	0.82	0.839	0.754
**3**	***B*. *pseudomallei* 1710b**	1	0.997	ID	0.994	0.994	0.914	0.908	0.909	0.914	0.912	0.995	0.91	0.961	0.925	0.906	0.906	0.906	0.906	0.905	0.909	0.909	0.909	0.895	0.834	0.821	0.828	0.82	0.842	0.755
**4**	***B*. *pseudomallei* BPC006**	0.994	0.997	0.994	ID	0.997	0.913	0.906	0.908	0.913	0.913	0.995	0.909	0.96	0.924	0.905	0.905	0.905	0.905	0.903	0.908	0.908	0.908	0.897	0.831	0.821	0.828	0.82	0.839	0.755
**5**	***B*. *pseudomallei* 1026b**	0.994	0.997	0.994	0.997	ID	0.912	0.905	0.906	0.912	0.912	0.995	0.908	0.958	0.923	0.903	0.903	0.903	0.903	0.902	0.906	0.906	0.906	0.895	0.829	0.82	0.827	0.818	0.838	0.755
**6**	***B*. *cepacia* GG4**	0.914	0.914	0.914	0.913	0.912	ID	0.965	0.957	0.965	0.898	0.913	0.941	0.92	0.946	0.96	0.954	0.96	0.961	0.962	0.968	0.954	0.968	0.897	0.843	0.831	0.828	0.829	0.85	0.754
**7**	***B*. *cenocepacia* J2315**	0.908	0.908	0.908	0.906	0.905	0.965	ID	0.958	0.976	0.902	0.906	0.945	0.916	0.939	0.95	0.957	0.968	0.947	0.949	0.956	0.938	0.95	0.897	0.828	0.831	0.831	0.829	0.853	0.753
**8**	***B*. *ambifaria* AMMD**	0.909	0.909	0.909	0.908	0.906	0.957	0.958	ID	0.962	0.901	0.908	0.935	0.914	0.938	0.946	0.971	0.957	0.947	0.941	0.951	0.934	0.943	0.895	0.839	0.825	0.827	0.823	0.851	0.737
**9**	***B*. *cenocepacia* MC0-3**	0.914	0.914	0.914	0.913	0.912	0.965	0.976	0.962	ID	0.905	0.913	0.936	0.916	0.949	0.95	0.961	0.982	0.951	0.947	0.956	0.938	0.946	0.903	0.836	0.834	0.832	0.832	0.861	0.754
**10**	***B*. *glumae* BGR1**	0.912	0.914	0.912	0.913	0.912	0.898	0.902	0.901	0.905	ID	0.914	0.903	0.91	0.914	0.888	0.906	0.901	0.882	0.891	0.888	0.891	0.894	0.92	0.828	0.818	0.817	0.816	0.84	0.75
**11**	***B*. *mallei* SAVP1**	0.995	0.998	0.995	0.995	0.995	0.913	0.906	0.908	0.913	0.914	ID	0.909	0.96	0.925	0.905	0.905	0.905	0.905	0.903	0.908	0.908	0.91	0.895	0.831	0.82	0.827	0.818	0.839	0.755
**12**	***B*. *multivorans* ATCC 17616**	0.91	0.91	0.91	0.909	0.908	0.941	0.945	0.935	0.936	0.903	0.909	ID	0.92	0.936	0.931	0.932	0.928	0.931	0.927	0.934	0.924	0.928	0.895	0.836	0.839	0.828	0.836	0.853	0.757
**13**	***B*. *thailandensis* E264**	0.961	0.961	0.961	0.96	0.958	0.92	0.916	0.914	0.916	0.91	0.96	0.92	ID	0.928	0.912	0.919	0.912	0.913	0.906	0.916	0.909	0.909	0.893	0.829	0.825	0.823	0.824	0.839	0.746
**14**	***B*. *vietnamiensis* G4**	0.925	0.925	0.925	0.924	0.923	0.946	0.939	0.938	0.949	0.914	0.925	0.936	0.928	ID	0.931	0.936	0.946	0.932	0.934	0.936	0.928	0.93	0.905	0.84	0.84	0.844	0.839	0.854	0.743
**15**	**Uncultured *Burkholderia* sp. HBadh-2**	0.906	0.906	0.906	0.905	0.903	0.96	0.95	0.946	0.95	0.888	0.905	0.931	0.912	0.931	ID	0.947	0.947	0.964	0.964	0.967	0.953	0.961	0.884	0.827	0.824	0.82	0.823	0.836	0.757
**16**	***B*. *ambifaria* MC40-6**	0.906	0.906	0.906	0.905	0.903	0.954	0.957	0.971	0.961	0.906	0.905	0.932	0.919	0.936	0.947	ID	0.961	0.947	0.949	0.958	0.936	0.946	0.903	0.844	0.835	0.832	0.832	0.854	0.743
**17**	***B*. *cenocepacia* HI2424**	0.906	0.906	0.906	0.905	0.903	0.96	0.968	0.957	0.982	0.901	0.905	0.928	0.912	0.946	0.947	0.961	ID	0.949	0.947	0.956	0.932	0.946	0.901	0.843	0.84	0.839	0.839	0.866	0.75
**18**	**Uncultured *Burkholderia* sp.HBadh-8**	0.906	0.906	0.906	0.905	0.903	0.961	0.947	0.947	0.951	0.882	0.905	0.931	0.913	0.932	0.964	0.947	0.949	ID	0.954	0.979	0.943	0.949	0.877	0.829	0.825	0.825	0.824	0.842	0.748
**19**	**Uncultured *Burkholderia* sp. HBadh-13**	0.905	0.905	0.905	0.903	0.902	0.962	0.949	0.941	0.947	0.891	0.903	0.927	0.906	0.934	0.964	0.949	0.947	0.954	ID	0.962	0.961	0.973	0.893	0.832	0.82	0.832	0.818	0.838	0.755
**20**	**Uncultured *Burkholderia* sp. HBadh-15**	0.909	0.909	0.909	0.908	0.906	0.968	0.956	0.951	0.956	0.888	0.908	0.934	0.916	0.936	0.967	0.958	0.956	0.979	0.962	ID	0.949	0.958	0.884	0.835	0.828	0.829	0.827	0.849	0.751
**21**	**Uncultured *Burkholderia* sp. HBadh-7**	0.909	0.909	0.909	0.908	0.906	0.954	0.938	0.934	0.938	0.891	0.908	0.924	0.909	0.928	0.953	0.936	0.932	0.943	0.961	0.949	ID	0.961	0.888	0.835	0.824	0.829	0.823	0.843	0.754
**22**	***B*. *lata* strain 383**	0.909	0.909	0.909	0.908	0.906	0.968	0.95	0.943	0.946	0.894	0.91	0.928	0.909	0.93	0.961	0.946	0.946	0.949	0.973	0.958	0.961	ID	0.894	0.84	0.824	0.825	0.823	0.84	0.759
**23**	***B*. *gladioli* BSR3**	0.895	0.895	0.895	0.897	0.895	0.897	0.897	0.895	0.903	0.92	0.895	0.895	0.893	0.905	0.884	0.903	0.901	0.877	0.893	0.884	0.888	0.894	ID	0.839	0.825	0.835	0.824	0.854	0.739
**24**	***B*. *xenovorans* LB400**	0.834	0.831	0.834	0.831	0.829	0.843	0.828	0.839	0.836	0.828	0.831	0.836	0.829	0.84	0.827	0.844	0.843	0.829	0.832	0.835	0.835	0.84	0.839	ID	0.877	0.872	0.875	0.923	0.717
**25**	***Burkholderia* sp. CCGE1001**	0.821	0.821	0.821	0.821	0.82	0.831	0.831	0.825	0.834	0.818	0.82	0.839	0.825	0.84	0.824	0.835	0.84	0.825	0.82	0.828	0.824	0.824	0.825	0.877	ID	0.901	0.997	0.891	0.711
**26**	***Burkholderia* sp. CCGE1003**	0.828	0.828	0.828	0.828	0.827	0.828	0.831	0.827	0.832	0.817	0.827	0.828	0.823	0.844	0.82	0.832	0.839	0.825	0.832	0.829	0.829	0.825	0.835	0.872	0.901	ID	0.899	0.875	0.714
**27**	***B*. *phenoliruptrix* BR3459a**	0.82	0.82	0.82	0.82	0.818	0.829	0.829	0.823	0.832	0.816	0.818	0.836	0.824	0.839	0.823	0.832	0.839	0.824	0.818	0.827	0.823	0.823	0.824	0.875	0.997	0.899	ID	0.888	0.71
**28**	***B*. *phytofirmans* PsJN**	0.842	0.839	0.842	0.839	0.838	0.85	0.853	0.851	0.861	0.84	0.839	0.853	0.839	0.854	0.836	0.854	0.866	0.842	0.838	0.849	0.843	0.84	0.854	0.923	0.891	0.875	0.888	ID	0.725
**29**	***B*. *phymatum* STM815**	0.755	0.754	0.755	0.755	0.755	0.754	0.753	0.737	0.754	0.75	0.755	0.757	0.746	0.743	0.757	0.743	0.75	0.748	0.755	0.751	0.754	0.759	0.739	0.717	0.711	0.714	0.71	0.725	ID

### Design of PCR primers and amplification of *Burkholderia bdha*

Of the 786 bp gene length, sites that amplify 730 bp were targeted for PCR amplification of *bdha* gene. The forward primer *bdha*-F was a 19-mer oligonucleotide spanning bases from 12 to 30, comprising the first conserved region of the gene. On the other hand, reverse primer *bdha*-R was a 20-mer spanning complementary bases from 721 to 740, which represents the conserved region situated at the 3’ end of the gene. Screening of the designed PCR primers for rate of specificity within the *Burkholderia* species provided unequivocal result in terms of primer match and expected amplicon length. Upon performing PCR, specific amplification of 730 bp was observed without any non-specificity. The developed PCR assay was found to be highly specific in identification of *Burkholderia* species (Table 1) and the results were in complete concurrence with that of *recA* PCR.

**Fig 3:**
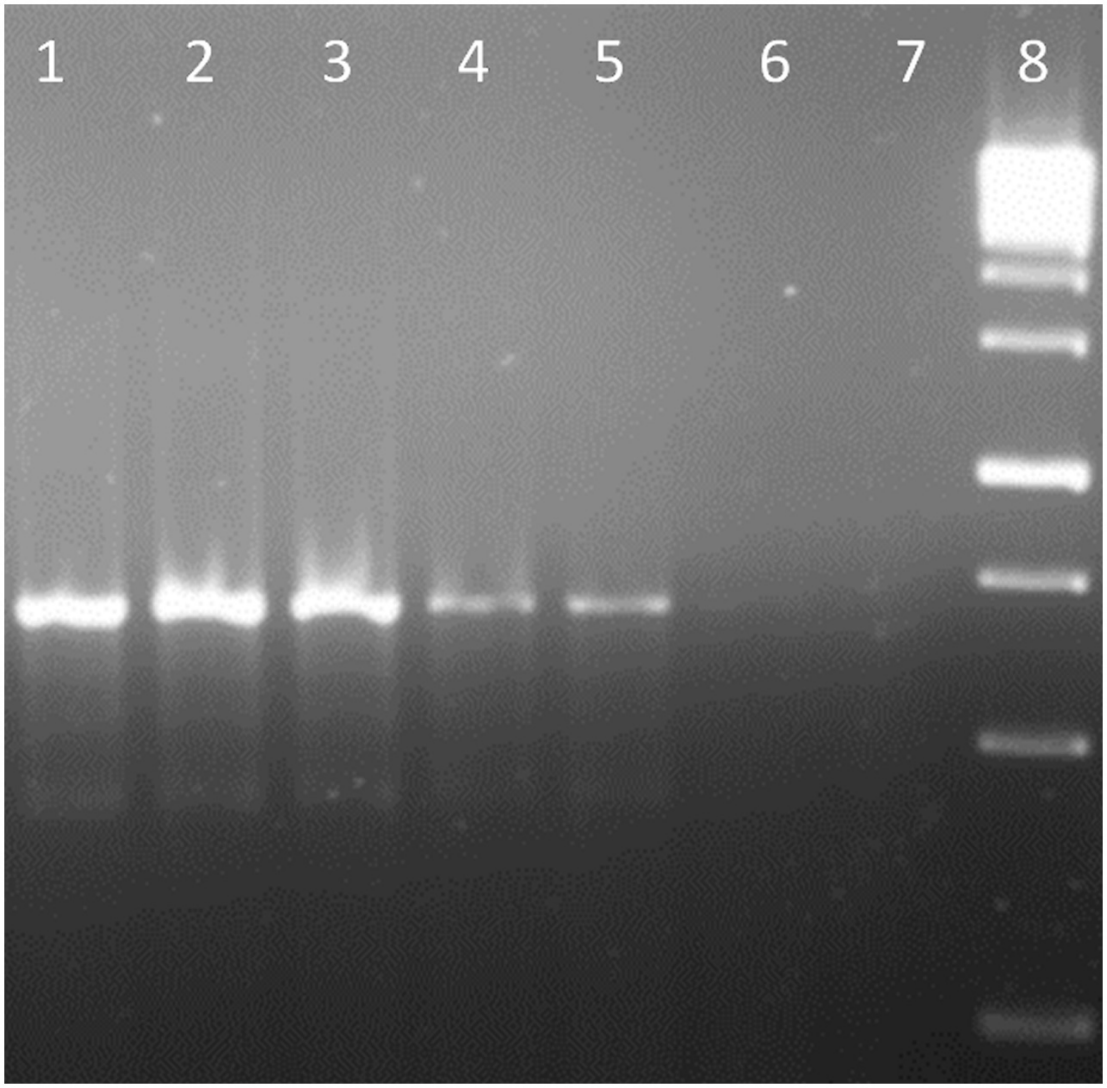
Limit of detection of the *bdha* PCR assay. Lane 1. 100 ng gDNA; Lane 2. 10 ng gDNA; Lane 3. 1 ng gDNA; Lane 4. 100 pg gDNA; Lane 5. 10 pg gDNA, Lane 6. 1 pg gDNA. Lane 7. 100 fg gDNA; Lane 8. 1 kb ladder.

Although *A*. *xylosoxidans* gave a strong BLASTn hit for the *bdha* gene of *B*. *pseudomallei*, the designed primer pair was sufficiently divergent from the *A*. *xylosoxidans* sequence. This *in silico* result correlated with a lack of amplification being detected in *A*. *xylosoxidans* using the *bdha* PCR. Meanwhile, screening for *bdha* sequence of closely related bacteria including *Pandoraea pnomenusa* and *P*. *aeruginosa* revealed that the designed primers were completely absent in them. In case of *R*. *solanacearum* only reverse primer was located with a mismatch at third nucleotide. Therefore, not surprisingly, the former mentioned bacterium was not identified in the developed *Burkholderia*-specific PCR assay. No reactivity was recorded with other non-*Burkholderia* strains as well. The specificity of the PCR was found to be reproducible. The analytical sensitivity of *bdha* PCR assay to detect *Burkholderia* was found to be 10 pg when evaluated for *B*. *pseudomallei* NCTC 10274 (Fig 3). However, *bdha* was found in duplicate copies, one copy on each chromosome, in case of *B*. *cepacia* GG4, *B*. *cenocepacia* strains H111, AU1054, J2315, *B*. *vietnamiensis* G4 and *B*. *gladioli* BSR3. Multiple copies of *bdha* in these species highlight the possible improvement in detection sensitivity of *bdha* PCR assay.

### Phylogenetic analysis of *Burkholderia bdha*, *recA* and 16S rDNA

**Fig 4:**
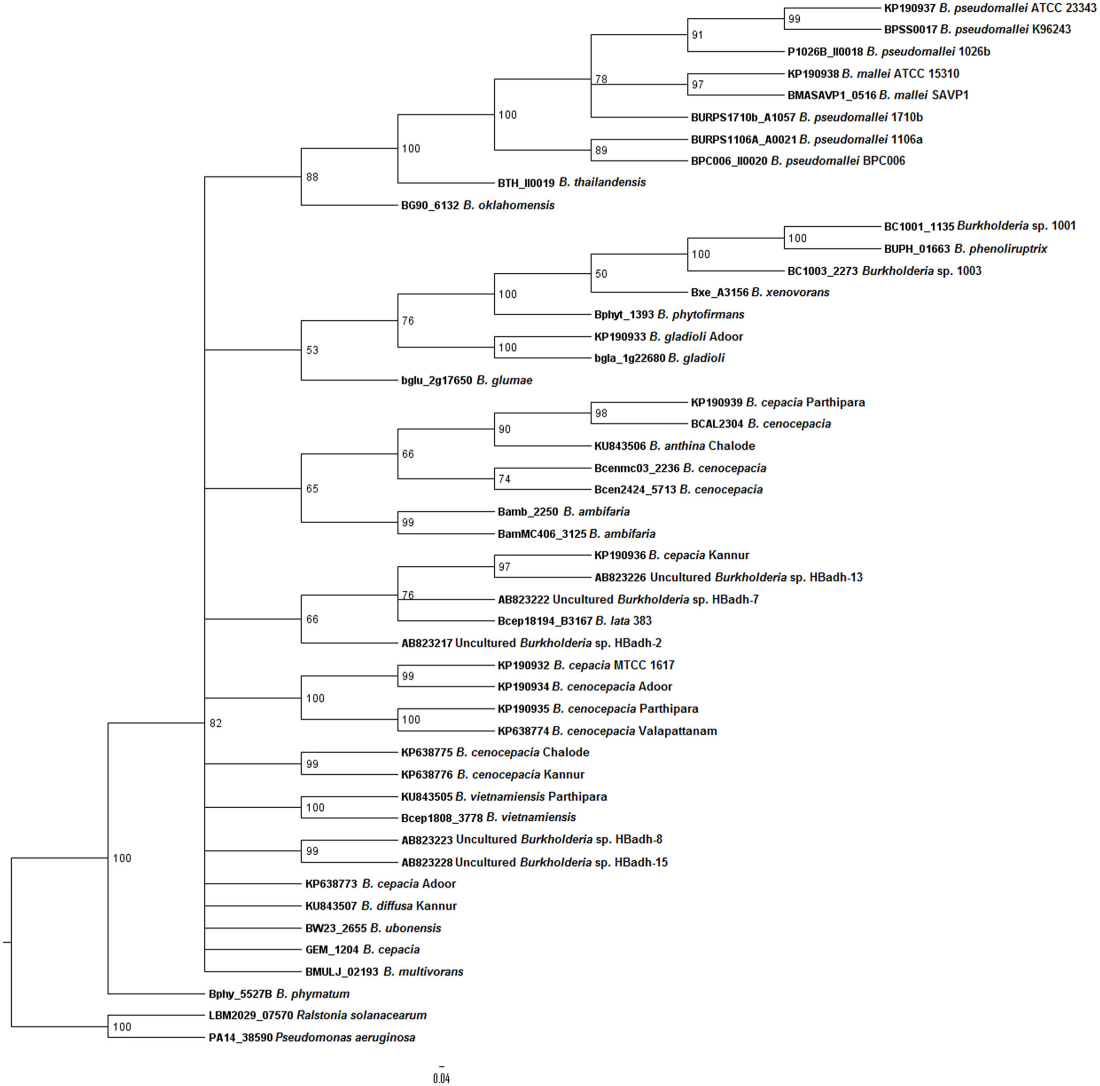
Phylogenetic analysis of environmental *Burkholderia* isolates from environmental samples in the Malabar coastal region of South India. A 730 bp region of *bdha* gene was sequenced and analyzed using Bayesian method. The phylogenetic tree is the consensus of 20000 trees and 50000 generations using the nucleotide substitution model GTR substitution model with gamma-distributed rate variation across sites and a proportion of invariable sites. Scale bar represents nucleotide substitutions per site.

**Fig 5:**
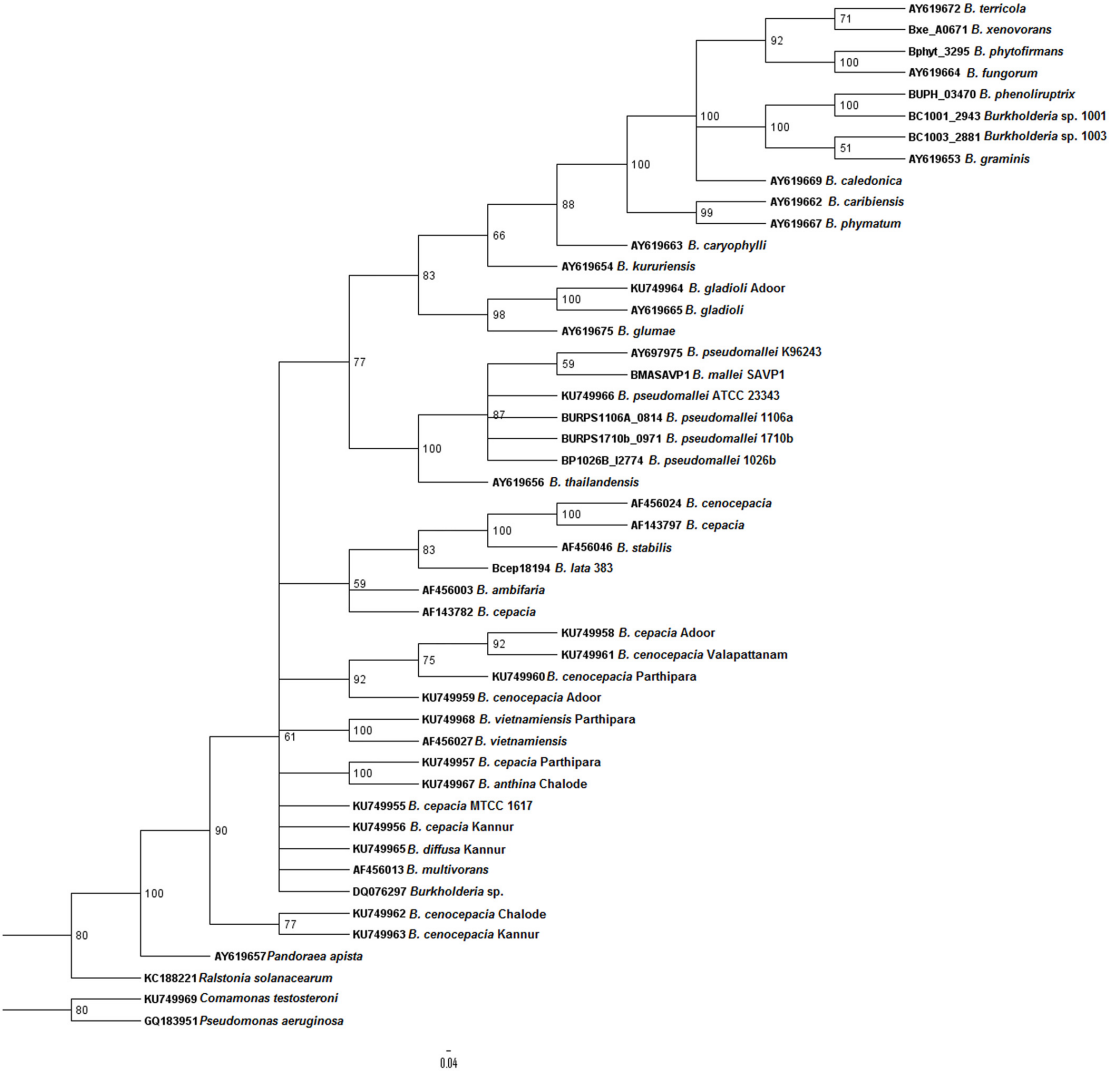
Phylogenetic analysis of environmental *Burkholderia* isolates from environmental samples in the Malabar coastal region of South India. A 869 bp region of *recA* gene was sequenced and analyzed using Bayesian method. The phylogenetic tree is the consensus of 20000 trees and 50000 generations using the nucleotide substitution model GTR substitution model with gamma-distributed rate variation across sites and a proportion of invariable sites. Scale bar represents nucleotide substitutions per site.

**Fig 6:**
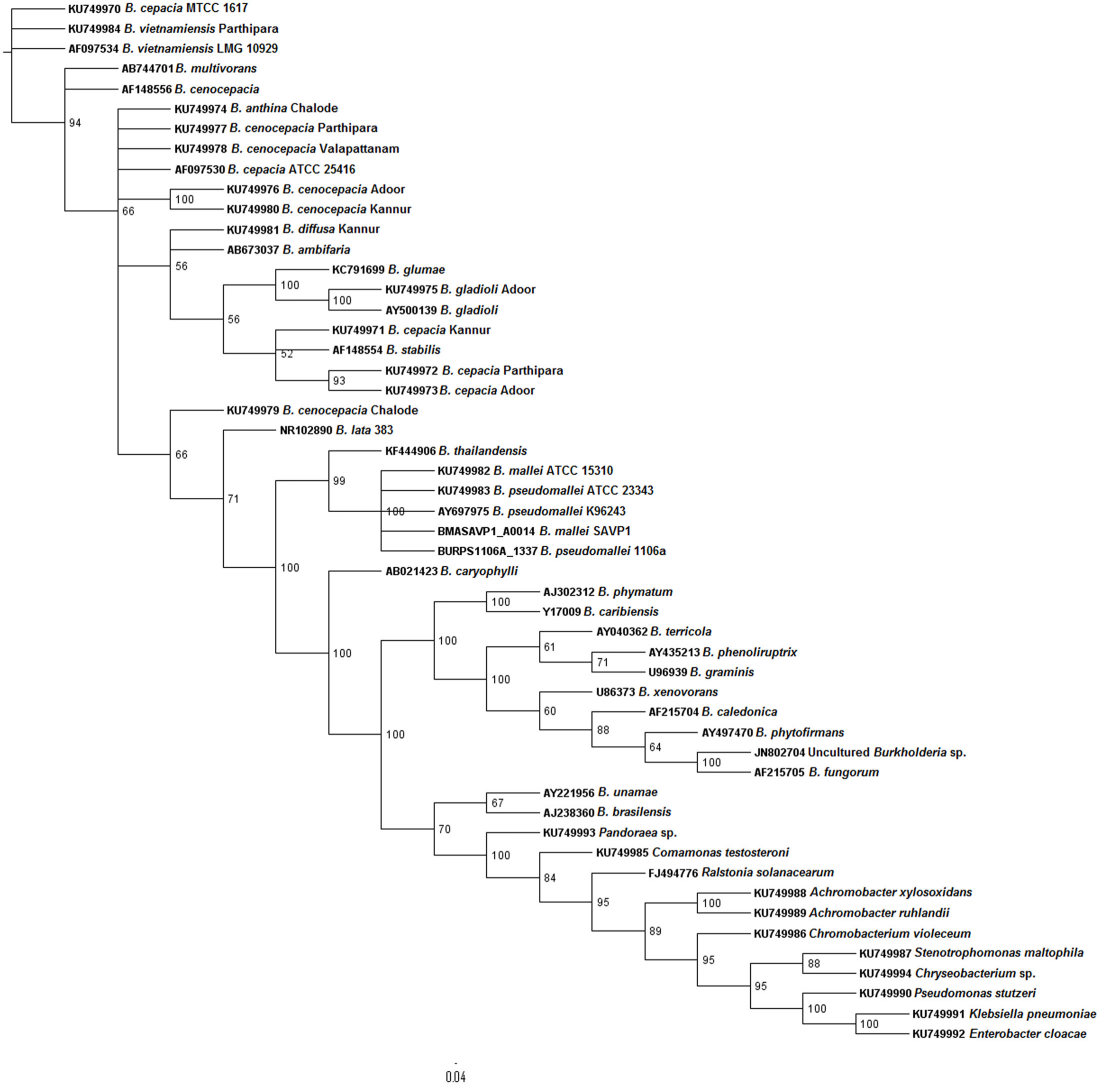
Phylogenetic analysis of environmental *Burkholderia* isolates from environmental samples in the Malabar coastal region of South India. A 1491 bp region of 16S rDNA gene was sequenced and analyzed using Bayesian method. The phylogenetic tree is the consensus of 20000 trees and 50000 generations using the nucleotide substitution model GTR substitution model with gamma-distributed rate variation across sites and a proportion of invariable sites. Scale bar represents nucleotide substitutions per site.

In order to determine the reliability of *bdha* gene in systematic identification of *Burkholderia* genus, we conducted phylogenetic analysis. The primary tool for better understanding of evolutionary relationships among organisms, *i*.*e*., phylogenetic tree was employed for this purpose. We followed a comparative approach to assess *bdha* gene. To achieve this, earlier established genes namely, *recA* and 16S rDNA were selected. Phylogenetic trees were constructed for all the three genes (Figs 4, 5 and 6). Bayesian analyses in case of all three genes studied revealed that the *Burkholderia* genus encompasses high diversity.

All three phylogeny trees revealed the distinct cluster of *B*. *cepacia* complex and *B*. *pseudomallei* group. The formation of distinct clusters—BCC and *B*. *pseudomallei* group revealed the possible co-evolution of the human pathogens. Though not highly distinct, *Burkholderia* species of agro-importance were also clustered as a separate group from the former mentioned groups. The clustering of *B*. *lata* strain 383 in the BCC group, in case of all three dendrograms, was observed. *B*. *phymatum* was found to be clustered separately from the saprophytic *Burkholderia* group in *bdha* phylogenetic tree. The insignificant e-value of the bacterium was ruled out as a reason for this difference in clustering. Due to the unavailability of *recA* sequence for uncultured *Burkholderia* species no information of their position in the genus was obtained. In the case of *bdha* topology uncultured *Burkholderia* species were found to be clustered within the BCC; whereas in case of 16S rDNA topology, they were grouped among *Burkholderia* species of agro-importance. *Burkholderia* sp. CGE1001 and CGE1003 were clustered in the plant related *Burkholderia* species in both *bdha* and *recA* topology. A different cluster was noticed for non-*Burkholderia* species namely *Ralstonia solanacearum*, *Pseudomonas aeruginosa*, *Chromobacterium* and *Pandoraea*.

## Discussion

This study has shown that *Burkholderia pseudomallei* and near-neighbour species occur in Malabar coastal region of Kerala. The prevalence of *B*. *pseudomallei* in the environmental samples of the region during rainy and winter season can be co-related with the cases of melioidosis in this region as reported in previous studies [25, 26, 27]. Presence of this bacterium in environment during rainy and wet seasons have been witnessed in endemic regions worldwide [5, 9, 37–42]. Diverse morphotypes demonstrated by the presumptive colonies on Ashdown agar were doubted as novel but unreported putative *B*. *pseudomallei* colony morphologies. Withal, detailed molecular characterization revealed that the isolates included only five *B*. *pseudomallei* strains with typical morphotypes illustrated by Chantratita and co-workers [43]. However, other morphotypes recovered from the samples collected this region were identified to be *Burkholderia* species belonging to *B*. *pseudomallei* group and BCC as well as non-*Burkholderia* species. The recovery of different species of *Burkholderia* using Ashdown medium, re-confirmed the broad range of selectivity confered by this culture medium [44, 45]. Though ASH medium is recommended for selective isolation of *B*. *pseudomallei* based on differential colony morphotyping, growth of non-*pseudomallei Burkholderia* and non-*Burkholderia* species, evidenced that the medium is not narrow in its range of selectivity. Similar results have been reported by other groups [46, 47].

Identification of *Burkholderia* strains among the isolates recovered in this study was done using *recA* PCR assay using specific primers BUR3 and BUR4. Encounter of *recA* negative results with some of the isolates was used as confirmation that the isolates included non-*Burkholderia* as well. 16S rDNA gene sequencing was adopted to identify those *recA* negative isolates. Parallely, all the *recA* positive isolates were subjected to both *recA* and 16S rDNA sequencing for identification of species. As a result, the bacterial consortium present in the regions studied was found to include *B*. *pseudomallei*, *B*. *thailandensis*, *B*. *cepacia*, *B*. *cenocepacia*, *B*. *anthina*, *B*. *ambifaria*, *B*. *diffusa* and *B*. *vietnamiensis* among *Burkholderia* and non-*Burkholderia* including *Comamonas testosteroni*, *Chromobacterium violaceum*, *Stenotrophomonas maltophilia*, *Achromobacter xylosoxidans*, *Achromobacter ruhlandii*, *Pseudomonas stutzeri*, *Klebsiella pneumoniae*, *Enterobacter cloacae*, *Pandoraea* sp. and *Chryseobacterium* sp. *B*. *pseudomallei* was successfully differentiated from *B*. *thailandensis* by performing arabinose utilization test as well as by multiplex PCR assay reported by Lee and co-workers [20].

In the process of determining highly conversed gene for detection and differentiation of *Burkholderia* species, emphasis was given to screen those involved in versatility and fundamental metabolic processes. Also, essential genes are more evolutionarily conserved than nonessential genes [48]. As a reason genes including oxidoreductases family, chaperones and structural protein genes were examined and *bdha* was found to be promising. *In silico* analysis of *bdha* for conservation and homology among *Burkholderia* species revealed a characteristic pattern of the arrangement of conservative regions. The conservative regions were found to be distributed in a mosaic pattern within the variable regions. This characteristic genetic distinction of *bdha* within the *Burkholderia* genus encouraged us for the development of *Burkholderia* genus-specific PCR assay. The novel pair of universal primer designed targeting conserved regions of the *bdha* resulted in a 730 bp PCR amplicon. The analytical sensitivity of our *bdha* PCR assay to detect *Burkholderia* was found to be 10 pg/μl when evaluated on *B*. *pseudomallei* NCTC 10274. However, *bdha* is present in duplicate copies, one copy on each chromosome, in case of *B*. *cepacia* GG4, *B*. *cenocepacia* strains H111, AU1054, J2315, *B*. *vietnamiensis* G4 and *B*. *gladioli* BSR3. Multiple copies of *bdha* in former mentioned species highlight the possible improvement in detection sensitivity of our PCR assay. This also explains the usefulness of the described PCR assay in sensitive detection of *Burkholderia*. Presence of *bdha* in *A*. *xylosoxidans* with 83% identity during BLASTN analysis was not a surprising issue. *A*. *xylosoxidans* is a beta proteobacterial pathogen often misidentified as *B*. *cepacia* complex in cystic fibrosis cases [49]. Absence of the designed primers for specific amplification of *bdha* in non-*Burkholderia* strains including *A*. *xylosoxidans*, *R*. *solanacearum*, *P*. *aeruginosa* revealed the detection specificity of the developed PCR assay.

Genus-specific PCR assays such as the novel *bdha* PCR play critical role in preliminary confirmation of outbreaks as well as endemicity of the continuously growing *Burkholderia* genus in a particular location. Due to the unavailability of representative strains of all *Burkholderia* species, we also retrieved their respective *bdha* sequences from NCBI database. Screening of our universal primers for rate of specificity within the *Burkholderia* species provided unequivocal result in terms of primer match and expected amplicon length. The sequence analysis of the 730 bp *bdha* portion revealed that sequence variations among *Burkholderia* species ranged from 8% to 29%. Hence higher level of sequence variations in *bdha* sequence confers more possibilities for confirmative distinction among *Burkholderia* species.

Earlier studies provide a molecular insight on the dual role of *bdha* in *Burkholderia*—promoting symbiosis with host plants and favoring the persistence of pathogenic *Burkholderia* under oxygen-limited conditions within the host [48, 50]. Yet, to the best of our knowledge, no studies have been implicated on the possible role of *bdha* gene in evolution of bacteria. Molecular evolutionary studies comprising of genome sequence analysis and knockout experiments on *Escherichia coli*, *Helicobacter pylori* and *Neisseria meningitidis* revealed that essential genes of bacteria are more conserved than nonessential genes over microevolutionary and macroevolutionary time scales [51]. On this basis, we evaluated *bdha* gene for possible diagnostic and phylogenetic application with respect to *Burkholderia* species. To achieve this, phylogenetic trees were constructed based on *recA*, 16S rDNA and *bdha* sequences, respectively (Figs 4, 5 and 6). The comparison of phylogenetic trees was robust since the tree-splits were treated differentially based on the bootstrap value.

Several groups have performed phylogenetic studies on *Burkholderia* species, mainly on BCC using *recA* [13, 15] and16S rDNA [12] genes. We took major care to consider almost all species and strains of *Burkholderia* used in the previous studies and performed phylogenetic study for their respective *bdha*, *recA* and 16S rDNA afresh. Uncultured *Burkholderia* species data was also considered based on feasibility and availability of required gene sequence information. We made additional improvements in the present phylogenetic study by involving more than one strain of species wherever possible in order to obtain consistent results. Bayesian analysis, the most recommended method to elucidate the patterns of pathogens dispersal in a phylogeny, was employed in order to attain accuracy in evaluation of uncertainty of phylogenies [52]. Bayesian reconstruction methods also enable further generalization of conditional probability analysis by removing the necessity to fix the Markov model parameters to obtain ancestral states and the necessity to specify a fixed tree topology with known branch lengths. Bayesian inference integrates conclusions over all possible parameter values [53]. Upon comparison, all three genes resulted in congruent separation of *Burkholderia* species into two major clusters, one comprised of *B*. *pseudomallei* group and the other comprised of BCC. Both *bdha* and *recA* based phylogenetic trees resulted in tight and differentiable clustering of Bcc members and *B*. *pseudomallei* group. Clustering of *B*. *lata* strain 383 was noteworthy in all three trees. *B*. *lata* strain 383 is a member of BCC commonly encountered as an industrial contaminant with inherent resistance to preservatives [54]. Clustering of uncultured *Burkholderia* species within the BCC cluster in case of *bdha* phylogeny provides valuable information that they are possibly co-evolved along with the BCC members or they are members of the complex. This signified the discriminatory proficiency of *bdha* in terms of evolution of *Burkholderia* species in a precise way similar to *recA*. These observations indicate that the use of the *bdha* gene of the present study stands as an unequivocal strategy with bilateral application of being exercised as a specific detection tool and evolution analyzer.

In conclusion, we studied the prevalence of *B*. *pseudomallei* and its near-neighbor species in the Malabar coastal region of South India. The prevalence of these pathogenic species in the region highlights the possibility of exposure of the habitats to these infectious agents. Besides to the prevalence study, we also have established a novel, user friendly and deployable PCR method followed by sequencing of PCR amplicon for the detection of biologically significant species of *Burkholderia*. The results indicate the applicability of our PCR method followed by the sequencing of *bdha* PCR amplicon in microbial detection from environmental and clinical samples during any outbreaks or in routine laboratory diagnoses. Besides nucleotide sequencing approach, the inbuilt interspecies variations with respect to *bdha* might possibly promote for the development of species-specific internal probes for identification of *Burkholderia* at species level. The species-specific internal probes can be employed in several DNA based detection platforms including real-time PCR, microarrays and oligonucleotide arrays for identification of *Burkholderia* species. As a possible advantage, one need not explore specific genes for each individual species separately. The clustering of the *Burkholderia* species in case of *bdha* phylogenetic tree, though in general agreement with that of *recA* and 16S rDNA trees, noteworthy clustering of uncultured *Burkholderia* species within BCC highlights the discriminatory power of *bdha*. Based on our study, future developments of species-specific detection assays based on the *bdha* sequence polymorphisms or use of the gene as a component of multiple-locus sequence type database for pertinent use as molecular diagnostic tool are foreseen.

## Supporting Information

S1 Supporting Information Bacterial strains employed for *recA*, *bdha* and 16S *rRNA* phylogenetic analysis. (DOCX)
