# Supplementary material for: Prevalence and Identification of Burkholderia pseudomallei and Near-Neighbor Species in the Malabar Coastal Region of India
Source: PLoS Negl Trop Dis. 2016 Sep 15;10(9):e0004956. doi: 10.1371/journal.pntd.0004956 (PMC5025242; doi:10.1371/journal.pntd.0004956)
Supplement: S1 Supporting Information — (DOCX) [file pntd.0004956.s001.docx]

**Supporting Information 1:**

**Bacterial strains employed for *recA*, *bdha* and 16S *rRNA* phylogenetic analysis**

| **Bacterial strains employed for *recA* phylogenetic analysis** |
| --- |
| >KU749955 Seq1_MTCC 1617 *Burkholderia cepacia*_recA partial cds |
| >KU749956 Seq2_Kannur *Burkholderia cepacia* isolate_recA partial cds |
| >KU749957 Seq3_Parthipara *Burkholderia cepacia* isolate_recA partial |
| >KU749958 Seq4_Adoor *Burkholderia cepacia* isolate_recA partial cds |
| >KU749959 Seq5_Adoor *Burkholderia cenocepacia* isolate_recA partial cds |
| >KU749960 Seq6_Parthipara *Burkholderia cenocepacia* isolate_recA |
| >KU749961 Seq7_Valapattanam *Burkholderia cenocepacia* isolate_recA |
| >KU749962 Seq8_Chalode *Burkholderia cenocepacia* isolate_recA partial |
| >KU749963 Seq9_Kannur *Burkholderia cenocepacia* isolate_recA partial |
| >KU749964 Seq10_Adoor *Burkholderia gladioli* isolate_recA partial cds |
| >KU749965 Seq11_Kannur *Burkholderia diffusa* isolate_recA partial cds |
| >KU749966 Seq12_*Burkholderia pseudomallei* ATCC 23343_recA partial cds |
| >KU749967 Seq13_Chalode *Burkholderia anthina* isolate_recA partial cds |
| >KU749968 Seq14_Parthipara *Burkholderia vietnamiensis* isolate_recA |
| >KU749969 *Comamonas testosteroni* 1001 |
| >AY619672 *Burkholderia terricola* |
| >AY619662 *Burkholderia caribiensis* |
| >BURPS1106A_0814 *Burkholderia pseudomallei* 1106a |
| >BURPS1710b_0971 *Burkholderia pseudomallei* 1710b |
| >BP1026B_I2774 *Burkholderia pseudomallei* 1026b |
| >AY697975 *Burkholderia pseudomallei* K96243 |
| >AF456024 *Burkholderia cenocepacia* C1714 |
| >AF456003 *Burkholderia ambifaria* ATCC 53266 |
| >AY619665 *Burkholderia gladioli* LMG 2216 |
| >AY619675 *Burkholderia glumae* 2196 |
| >BMASAVP1 *Burkholderia mallei* SAVP1 |
| >AF456013 *Burkholderia multivorans* C1524 |
| >BUPH_03470 *Burkholderia phenoliruptrix* BR3459a |
| >Bphyt_3295 *Burkholderia phytofirmans* PsJN |
| >Bcep18194 *Burkholderia lata* 383 |
| >BC1001_2943 *Burkholderia* sp. CCGE1001 |
| >BC1003_2881 *Burkholderia* sp. CCGE1003 |
| >AY619656 *Burkholderia thailandensis* LMG 20219 |
| >AF456027 *Burkholderia vietnamiensis* ATCC 29424 |
| >Bxe_A0671 *Burkholderia xenovorans* LB400 |
| >AF143782 *Burkholderia cepacia* C4455 |
| >AF143797 *Burkholderia cepacia* M36 |
| >AF456046 *Burkholderia stabilis* HI-2462 |
| >AY619653 *Burkholderia graminis* C4D1M LMG 18924 |
| >AY619664 *Burkholderia fungorum* LMG 16225 |
| >AY619663 *Burkholderia caryophylli* LMG 2155 |
| >AY619667 *Burkholderia phymatum* STM815 LMG 21445 |
| >DQ076297 *Burkholderia* sp. clone 2a1CL3.4 |
| >AY619654 *Burkholderia kururiensis* LMG 19447 |
| >AY619669 *Burkholderia caledonica* LMG 19076 |
| >KC188221 *Ralstonia solanacearum* |
| >AY619657 *Pandoraea apista* |
| >GQ183951 *Pseudomonas aeruginosa* |

| **Bacterial strains employed for *bdha* phylogenetic analysis** |
| --- |
| >KP190932 *Burkholderia cepacia* MTCC 1617 |
| >KP190933 *Burkholderia gladioli* Adoor |
| >KP190934 *Burkholderia cenocepacia* Adoor |
| >KP190935 *Burkholderia cenocepacia* Parthipara |
| >KP190936 *Burkholderia cepacia* Kannur |
| >KP190937 *Burkholderia pseudomallei* ATCC 23343 |
| >KP190938 *Burkholderia mallei* ATCC 15310 |
| >KP190939 *Burkholderia cepacia* Parthipara |
| >KP638773 *Burkholderia cepacia* Adoor |
| >KP638774 *Burkholderia cenocepacia* Valapattanam |
| >KP638775 *Burkholderia cenocepacia* Chalode |
| >KP638776 *Burkholderia cenocepacia* Kannur |
| >KU843505 *Burkholderia vietnamiensis* Parthipara |
| >KU843506 *Burkholderia anthina* Chalode |
| >KU843507 *Burkholderia diffusa* Kannur |
| >Bphy_5527 *Burkholderia phymatum* STM815 |
| >BURPS1106A_A0021 *Burkholderia pseudomallei* 1106a |
| >BW23_2655 *Burkholderia ubonensis* MSMB22 |
| >BG90_6132 *Burkholderia oklahomensis* C6786 |
| >BURPS1710b_A1057 *Burkholderia pseudomallei* 1710b |
| >BPC006_II0020 *Burkholderia pseudomallei* BPC006 |
| >P1026B_II0018 *Burkholderia pseudomallei* 1026b |
| >BPSS0017 *Burkholderia pseudomallei* K96243 |
| >GEM_1204 *Burkholderia cepacia* GG4 |
| >BCAL2304 *Burkholderia cenocepacia* J2315 |
| >Bamb_2250 *Burkholderia ambifaria* AMMD |
| >Bcenmc03_2236 *Burkholderia cenocepacia* MC0-3 |
| >bglu_2g17650 *Burkholderia glumae* BGR1 |
| >BMASAVP1_0516 *Burkholderia mallei* SAVP1 |
| >BMULJ_02193 *Burkholderia multivorans* ATCC 17616 |
| >BTH_II0019 *Burkholderia thailandensis* E264 |
| >Bcep1808_3778 *Burkholderia vietnamiensis* G4 |
| >AB823217 Uncultured *Burkholderia* sp. HBadh-2 |
| >BamMC406_3125 *Burkholderia ambifaria* MC40-6 |
| >Bcen2424_5713 *Burkholderia cenocepacia* HI2424 |
| >AB823223 Uncultured *Burkholderia* sp.HBadh-8 |
| >AB823226 Uncultured *Burkholderia* sp. HBadh-13 |
| >AB823228 Uncultured *Burkholderia* sp. HBadh-15 |
| >AB823222 Uncultured *Burkholderia* sp. HBadh-7 |
| >Bcep18194_B3167 *Burkholderia lata* 383 |
| >bgla_1g22680 *Burkholderia gladioli* BSR3 |
| >Bxe_A3156 *Burkholderia xenovorans* LB400 |
| >BC1001_1135 *Burkholderia* sp. CCGE1001 |
| >BC1003_2273 *Burkholderia* sp. CCGE1003 |
| >BUPH_01663 *Burkholderia phenoliruptrix* BR3459a |
| >Bphyt_1393 *Burkholderia phytofirmans* PsJN |
| >LBM2029_07570 *Ralstonia solanacearum* |
| >PA14_38590 *Pseudomonas aeruginosa* |

| **Bacterial strains employed for 16S *rRNA* phylogenetic analysis** |
| --- |
| >KU749970 MTCC 1617 *Burkholderia cepacia* |
| >KU749971 Kannur *Burkholderia cepacia* |
| >KU749972 Parthipara *Burkholderia cepacia* |
| >KU749973 Adoor *Burkholderia cepacia* |
| >KU749974 Chalode *Burkholderia anthina* |
| >KU749975 Adoor *Burkholderia gladioli* |
| >KU749976 Adoor *Burkholderia cenocepacia* |
| >KU749977 Parthipara *Burkholderia cenocepacia* |
| >KU749978 Valapattanam *Burkholderia cenocepacia* |
| >KU749979 Chalode *Burkholderia cenocepacia* |
| >KU749980 Kannur *Burkholderia cenocepacia* |
| >KU749981 Kannur *Burkholderia diffusa* |
| >KU749982 *Burkholderia mallei* ATCC 15310 |
| >KU749983 *Burkholderia pseudomallei* ATCC 23343 |
| >KU749984 Parthipara *Burkholderia vietnamiensis* |
| >KU749985 *Comamonas testosteroni* 1001 |
| >KU749986 *Chromobacterium violeceum* |
| >KU749987 *Stenotrophomonas maltophila* 1002 |
| >KU749988 *Achromobacter xylosoxidans* 1003 |
| >KU749989 *Achromobacter ruhlandii* 1004 |
| >KU749990 *Pseudomonas stutzeri* 1005 |
| >KU749991 *Klebsiella pneumonia* 1006 |
| >KU749992 *Enterobacter cloacae* 1007 |
| >KU749993 *Pandoraea* sp. 1008 |
| >KU749994 *Chryseobacterium* sp. 1009 |
| >FJ494776 *Ralstonia solanacearum* |
| >JN802704 Uncultured *Burkholderia sp.* |
| >NR102890 *Burkholderia lata* 383 |
| >KC791699 *Burkholderia glumae* |
| >AB744701 *Burkholderia multivorans* |
| >AY435213 *Burkholderia phenoliruptrix* |
| >AY500139 *Burkholderia gladioli* |
| >AB673037 *Burkholderia ambifaria* |
| >AY697975 *Burkholderia pseudomallei* K96243 |
| >AF148556 *Burkholderia cenocepacia* |
| >BMASAVP1_A0014 *Burkholderia mallei* |
| >BURPS1106A_1337 *Burkholderia pseudomallei* 1106a |
| >KF444906 *Burkholderia thailandensis* |
| >U86373 *Burkholderia xenovorans* |
| >AF215704 *Burkholderia caledonica* LMG 19076 |
| >AY497470 *Burkholderia phytofirmans* PsJN |
| >AF215705 *Burkholderia fungorum* LMG 16225 |
| >AY040362 *Burkholderia terricola* |
| >U96939 *Burkholderia graminis* |
| >AJ302312 *Burkholderia phymatum* |
| >Y17009 *Burkholderia caribiensi* |
| >AY221956 *Burkholderia unamae* MTl-641 |
| >AJ238360 *Burkholderia brasilensis* |
| >AB021423 *Burkholderia caryophylli* |
| >AF148554 *Burkholderia stabilis* |
| >AF097530 *Burkholderia cepacia* ATCC 25416 |
| >AF097534 *Burkholderia vietnamiensis* LMG 10929 |
